# The role of preoperative diffusion tensor imaging in predicting and improving functional outcome in pediatric patients undergoing epilepsy surgery: a systematic review

**DOI:** 10.1259/bjro.20200002

**Published:** 2021-07-05

**Authors:** Jose Leon-Rojas, Isabel Cornell, Antonio Rojas-Garcia, Felice D’Arco, Jasmina Panovska-Griffiths, Helen Cross, Sotirios Bisdas

**Affiliations:** 1 Department of Neuroradiology, The National Hospital for Neurology and Neurosurgery, University College London Hospitals NHS Trust, London, UK; 2 NeurALL Research Group, Universidad Internacional del Ecuador, Medical School, Quito, Ecuador; 3 Department of Applied Health Research, University College London, London, UK; 4 Department of Pediatric Neuroradiology, Great Ormond Street Hospital for Children NHS Trust, London, UK; 5 Department of Brain Repair and Rehabilitation, Institute of Neurology, University College London, London, UK

## Abstract

**Objective:**

Diffusion tensor imaging (DTI) is a useful neuroimaging technique for surgical planning in adult patients. However, no systematic review has been conducted to determine its utility for pre-operative analysis and planning of Pediatric Epilepsy surgery. We sought to determine the benefit of pre-operative DTI in predicting and improving neurological functional outcome after epilepsy surgery in children with intractable epilepsy.

**Methods:**

A systematic review of articles in English using PubMed, EMBASE and Scopus databases, from inception to January 10, 2020 was conducted. All studies that used DTI as either predictor or direct influencer of functional neurological outcome (motor, sensory, language and/or visual) in pediatric epilepsy surgical candidates were included. Data extraction was performed by two blinded reviewers. Risk of bias of each study was determined using the QUADAS 2 Scoring System.

**Results:**

13 studies were included (6 case reports/series, 5 retrospective cohorts, and 2 prospective cohorts) with a total of 229 patients. Seven studies reported motor outcome; three reported motor outcome prediction with a sensitivity and specificity ranging from 80 to 85.7 and 69.6 to 100%, respectively; four studies reported visual outcome. In general, the use of DTI was associated with a high degree of favorable neurological outcomes after epilepsy surgery.

**Conclusion:**

Multiple studies show that DTI helps to create a tailored plan that results in improved functional outcome. However, more studies are required in order to fully assess its utility in pediatric patients. This is a desirable field of study because DTI offers a non-invasive technique more suitable for children.

**Advances in knowledge:**

This systematic review analyses, exclusively, studies of pediatric patients with drug-resistant epilepsy and provides an update of the evidence regarding the role of DTI, as part of the pre-operative armamentarium, in improving post-surgical neurological sequels and its potential for outcome prediction.

## Introduction

### Pediatric epilepsy

According to the current guidelines of the International League Against Epilepsy (ILAE), epilepsy is defined as disease of the brain characterized by the presence of at least two unprovoked seizures happening more than 24 h apart, or one unprovoked seizure with a likelihood of more than 60% of having another one, or an established diagnosis of an epilepsy syndrome.^1^ Pediatric epilepsy has a prevalence of 5.5–8.8 per 1000 person-years in resource rich settings,^2,3^ while in developing countries the numbers are much higher reaching 10.2 per 1000 person-years.^4^ Furthermore, children diagnosed with epilepsy have a higher risk of suffering from other conditions such as anxiety, depression, attention-deficit/hyperactivity disorder, problems with conduct, developmental delay and even poor social competence.^3^ In the majority of cases, epilepsy can be safely controlled with medication, however in around 30% of cases, the condition is resistant to pharmacological treatment (*i.e.* refractory epilepsy).^5^ Indeed, the ILAE defines it as drug-resistant epilepsy, characterized by the failure to obtain seizure freedom with two antiepileptic drugs (AED) either as monotherapy or in combination.^6^

### The role of epilepsy surgery

Previous research^3^ has suggested that children with epilepsy have a higher risk of long-term neurodevelopmental impairment as well as poor psychological and social outcomes; these risks become even more significant when we consider the aforementioned group of children who do not respond to AED.^3,7^ A resolution in these cases involves the surgical resection of the brain area where the aberrant electrical discharges are originating (*i.e.* the epileptogenic region). Surgical resection for pediatric drug-resistant epilepsy has a clear benefit as shown by a randomized controlled trial by Dwivedi and collaborators in 2017 that randomly allocated 116 pediatric patients to either a surgery with medical treatment group or to a medical treatment only group. At 12 months, 77% of the patients from the surgery group were completely seizure-free compared to 7% in the medical treatment group only.^7^ However, there is a fine balance between the risks and benefits of such procedure, especially when the epileptogenic foci is located in areas of the brain that contain important white matter tracts (WMT) that are involved in vital functions such as language, vision, motor function and sensation.^8^ Therefore, knowledge of the location of these tracts is vital to perform a safe surgical resection and guarantee optimal post-surgical neurological outcome.^8,9^ Thankfully, such techniques do exist, based on the diffusion of water molecules within the WMT enabling their complete reconstruction and visualization in three-dimensions by using data from diffusion MRI.^10^

Multiple parameters obtained with this type of imaging have been used as an indirect measurement of WMT integrity such as fractional anisotropy (FA), mean diffusivity (MD), axial diffusivity (AD), and radial diffusivity (RD).^11^ Multiple studies, in both adults and pediatric patients have used these variables to describe the changes the epilepsy cause on WMT integrity.^12^ Certainly, such changes have been reported particularly in temporal lobe epilepsy.^12,13^ However, other studies have also reported that, in epileptic pediatric patients, changes in these values was minimal and with low variability, irrespective of epilepsy duration.^11^ Surgery in pediatric patients with intractable epilepsy often involves the resection of an epileptogenic lesion or area, that is often circumscribed and caused by non-malignant conditions, generating very little WMT microstructural changes.^14^ However, reorganization of the WMT and of the functional areas in pediatric patients’ brains have been widely reported in the literature.^15,16^ Therefore, a thorough pre-operative work-up should be performed, in order to properly demarcate the area to be excised and the associated eloquent areas that should be respected during surgery, by using multiple modalities such as scalp EEG, video EEG, fMRI, tractography, among others.^17^

### The role of diffusion tensor imaging in epilepsy surgery

**Figure 1.**
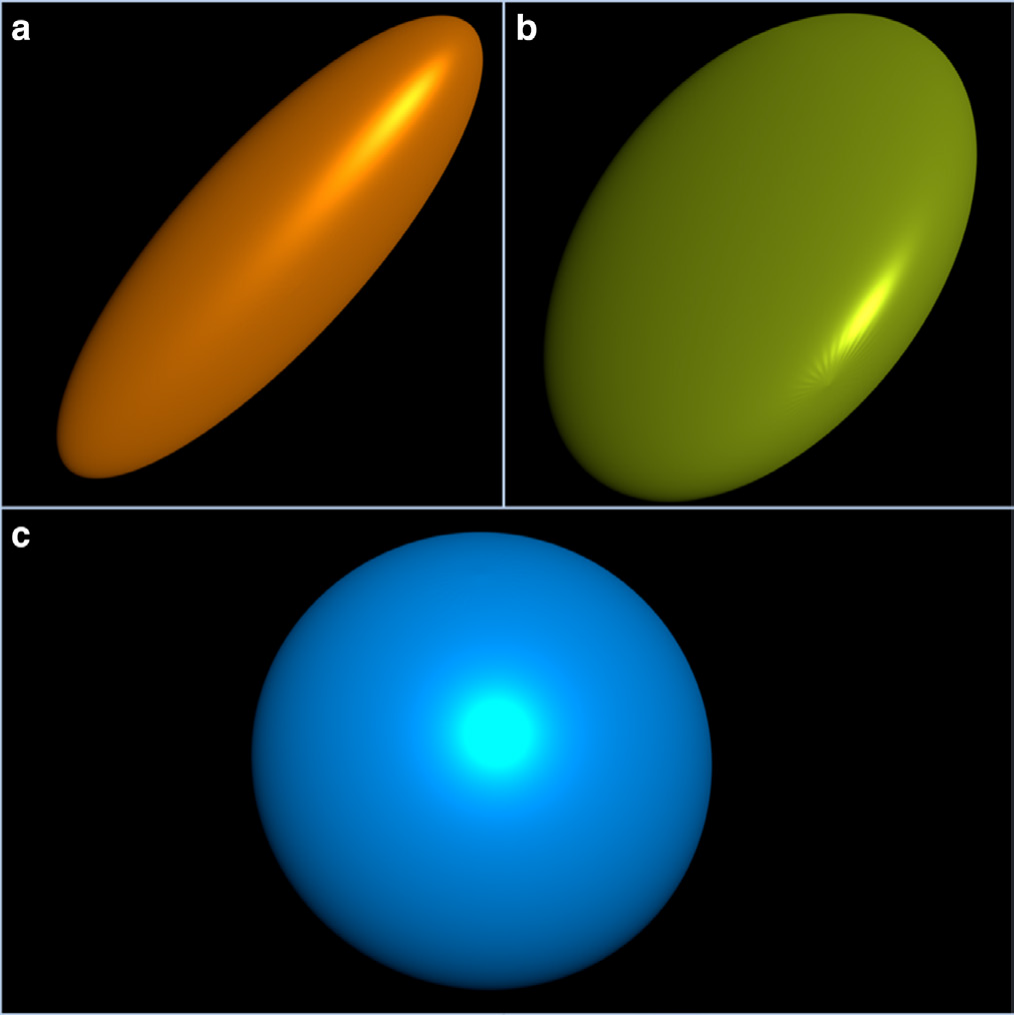
(a) Ellipsoid representing anisotropic diffusion of water molecules, also known as a Prolate Tensor. In this case, the relationship between Eigen values is: λ1 > λ2, λ3. (b) Oblate Tensor. The relationship between Eigen values is: λ1=λ2. (c). Spherical Tensor. The relationship between Eigen values is: λ1=λ2= λ3.

Diffusion-weighted imaging (DWI) and diffusion tensor imaging (DTI) are MRI sequences that are highly sensitive to diffusion of water molecules within the brain.^10,18^ This diffusion takes place in a 3D-space, therefore when water molecules move freely in all directions at the same rate, *e.g.* within the cerebrospinal fluid (CSF), the 3D shape that represents this type of diffusion will be a sphere and is called isotropic diffusion; in contrast, when diffusion is limited by cell membranes, *e.g.* as within the WMT, the movement becomes limited and forced in an specific direction making the 3D shape that represents this type of diffusion an ellipsoidal shape (anisotropic diffusion)^10^ (Figure 1). The main direction of anisotropic diffusion, which will most certainly be higher and well defined within the WMT, is usually colour-coded as red (left–right), green (anteroposterior) and blue (superior–inferior).^10,18^

All of these data are used for the reconstruction of WMT in a process known as tractography and is influenced by multiple parameters, such as acquisition, fiber identification/reconstruction, mathematical modelling, region of interest (ROI) and stopping thresholds.^14^ Amongst this, the most important parameters are related with data acquisition: the number of gradient directions and the B-Value.^10,14^ As these numbers increase, so does the spatial resolution and the precision of WMT reconstruction, as more diffusion data are available to increase accuracy and reduce false negatives.^9,10,14^ For example, the routinely used DTI uses ≤30 gradient directions and a B-value of 1000 s mm^−2^, whereas more advanced acquisitions involve a significantly higher number of gradient directions and B-values of >1000 s mm^−2^, in the case of High Angular Resolution Diffusion-Weighted Data (HARDI), or multiple B-values of 7000 s mm^−2^ in the case of Diffusion Spectrum Imaging (DSI).^14,19,20^

Finally, the mathematical model and the fiber reconstruction method are relevant as well, as they can be the difference between computing one fiber per voxel to multiple fibers per voxel and accurately dealing with fibers that come close together (kissing fibers) or that cross-each other (crossing fibers).^14,19^ The most used computational techniques to trace WMT are deterministic and probabilistic tractography.^9,10,14^ Deterministic tractography traces a WMT from a start to an end point in the brain and can only detect single fiber connections, meaning that it is significantly affected by the presence of crossing/kissing fibers within each voxel, often resulting in an inaccurate representation.^14,19,21^ In contrast, probabilistic tractography utilizes multiple iterations to determine the most likely path of the WMT by generating a map of connection probabilities meaning that it has a better signal-to-noise ratio and is less affected by crossing/kissing fibers.^14,19,20^

**Figure 2.**
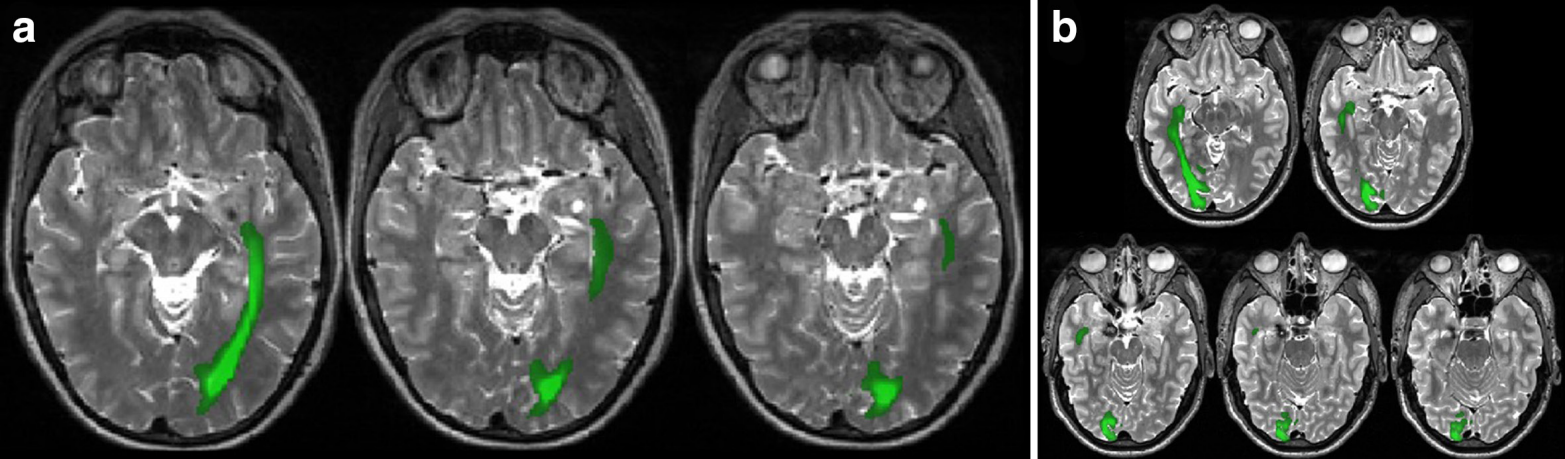
(a) Reconstructed right-sided optic radiation (colour-encoded in green) pathway using the source FA DTI data and probabilistic tractography methodology (FSL, University of Oxford, https://fsl.fmrib.ox.ac.uk/fsl/). The images were used for pre-operative resection planning of a right hippocampal cavernoma (arrow) in a 16-year-old male patient. (b) Reconstructed left-sided optic radiation (color-encoded in green) pathway using the source FA DTI data and probabilistic tractography methodology (FSL, University of Oxford, https://fsl.fmrib.ox.ac.uk/fsl/). The images were used for pre-operative resection planning in a 16-year-old male patient presenting with DNET in the left temporomesial region (arrows). DNET, dysembryoplastic neuroepithelial tumor; DTI, diffusion tensor imaging; FA, fractional anisotropy.

Regardless of the parameters used, the objective of tractography in epilepsy surgery is showing how WMT are related, in three-dimensional space, to the epileptogenic area as to prevent injury and reduce the surgical footprint in the brain.^22–24^ Figure 2 shows three pediatric case examples from the National Hospital of Neurology and Neurosurgery in which DTI was used to isolate the optic radiation (OR).

### Rationale and objectives

DWI and DTI have been used for multiple diseases in the adult population such as intracerebral hemorrhage,^25^ Parkinson’s disease,^26^ outcome prediction after stroke,^27^ among others; and even in the pediatric population, DTI has been used in diagnosis of brain malformations,^28^ and microstructural changes in preterm infants.^29^ The role of DTI and advanced tractography algorithms have been extensively assessed and proven effective in the adult population.^14,22,23^ However, a concise analysis of the literature reporting the use of DTI in pre-operative planning for pediatric epilepsy surgery is lacking. A systematic review of DTI in pediatric epilepsy by Szmuda et al. was published in 2016.^30^ However, although the study was referred to as pediatric, it incorporated studies with adult populations, *e.g.* of the six case–control studies they report,^31–36^ focusing on temporal lobe epilepsy and comprising of a total of 254 participants (both cases and controls), only 31 were under 19 years of age. Furthermore, their study didn’t report the number of included pediatric patients, but rather the number of included articles that interestingly also incorporated 11 review articles and 1 systematic review, a rather curious approach in a systematic review.^30^ Our work aims to specifically review the existing pediatric literature and summarize the use of DTI in children only (0–18 years-old) and illustrate how this imaging method modified the surgical plan, improved or predicted the functional outcome after epilepsy surgery.

## Methods and materials

The present systematic review was undertaken with reference to the Preferred Reporting Items for Systematic Reviews and Meta-Analyses (PRISMA): The PRISMA Statement.^37,38^ The protocol of this review was elaborated by following the checklist by the preferred reporting items for systematic review and meta-analysis protocols (PRISMA-P) 2015 statement^39^ and can be found in the appendix section. Additionally, it was registered in the international prospective register of systematic reviews (PROSPERO: https://www.crd.york.ac.uk/prospero/), ID: CRD42019120277.

### Eligibility criteria

A systematic review of the available scientific literature was conducted from inception to January 10, 2019 and only articles on peer-reviewed, indexed journals written in english were considered. Inclusion criteria for the selection of the studies were: (1) inclusion of pediatric patients (0–18 year-old) with diagnosis of intractable epilepsy, (2) who had undergone epilepsy surgery and (3) who had undergone DTI for pre-operative planning including localization of epileptogenic regions or lesions, localization of tracts to avoid injury or language lateralization. Exclusion criteria included: (1) articles whose majority of patients were adults or papers that didn’t allow for the extraction of only the pediatric population, (2) abstracts, letters to the editor, opinion articles or editorials, (3) literature or systematic reviews, (4) articles were the use of DTI was merely reported but its utility in the case was not clear. The articles had to report at least one functional outcome such as motor function, language function, visual function or somatosensory function. Data regarding post-surgical seizure-free status was also gathered when reported. All study designs were considered, including case reports as long as they met the inclusion and quality criteria.

### Information sources

Studies were identified by searching the following databases: PubMed (Inception-January 10, 2020), Ovid-EMBASE (Inception-January 10, 2020), and Scopus (Inception-January 10, 2020). No filters were applied on the searches and the last search was run in January 10, 2020. In addition, the author also searched for additional articles on the references of selected journal articles that were considered relevant to the current review. Screening of gray literature was not conducted for this review.

The following key terms were used to search the aforementioned databases: DTI, Diffusion Tensor Imaging, Preoperative, Surgery, Surgical, Operation, Neurosurgery, Epilepsy, Seizure, Convulsion, Ped*,Child*,Pediatric with a combination of the Boolean Terms “AND” and “OR”. The full search terms used in each database can be found in the Appendix.

### Study selection

Studies were selected using the aforementioned inclusion and exclusion criteria, independently by two reviewers***,*** firstly by looking at the abstracts and a second screening was performed by reading the full text of the remaining articles. The selection of articles was performed completely and independently by the two reviewers and any disagreements were resolved by discussion and consensus. Patients included in our analysis included only those younger than 19 years of age. If a study reported older patients, they were excluded from analysis. Data were independently extracted by the two blinded reviewers using and excel spreadsheet of predefined categories that needed to be sought in each paper. The categories included: study design, sample size, gender, age, imaging modalities used and parameters, clinical/pathological diagnosis, main findings, motor outcome, language outcome, sensory outcome, visual outcome and post-surgical seizure freedom. Data were compared afterwards, and any discrepancies were solved by discussion and consensus. As significant heterogeneity was noticed, the results will be analyzed in a qualitative and descriptive manner. All the articles were managed in the Mendeley Reference Manager Software, that was also utilized to remove duplicates.

### Risk of bias

**Figure 3.**
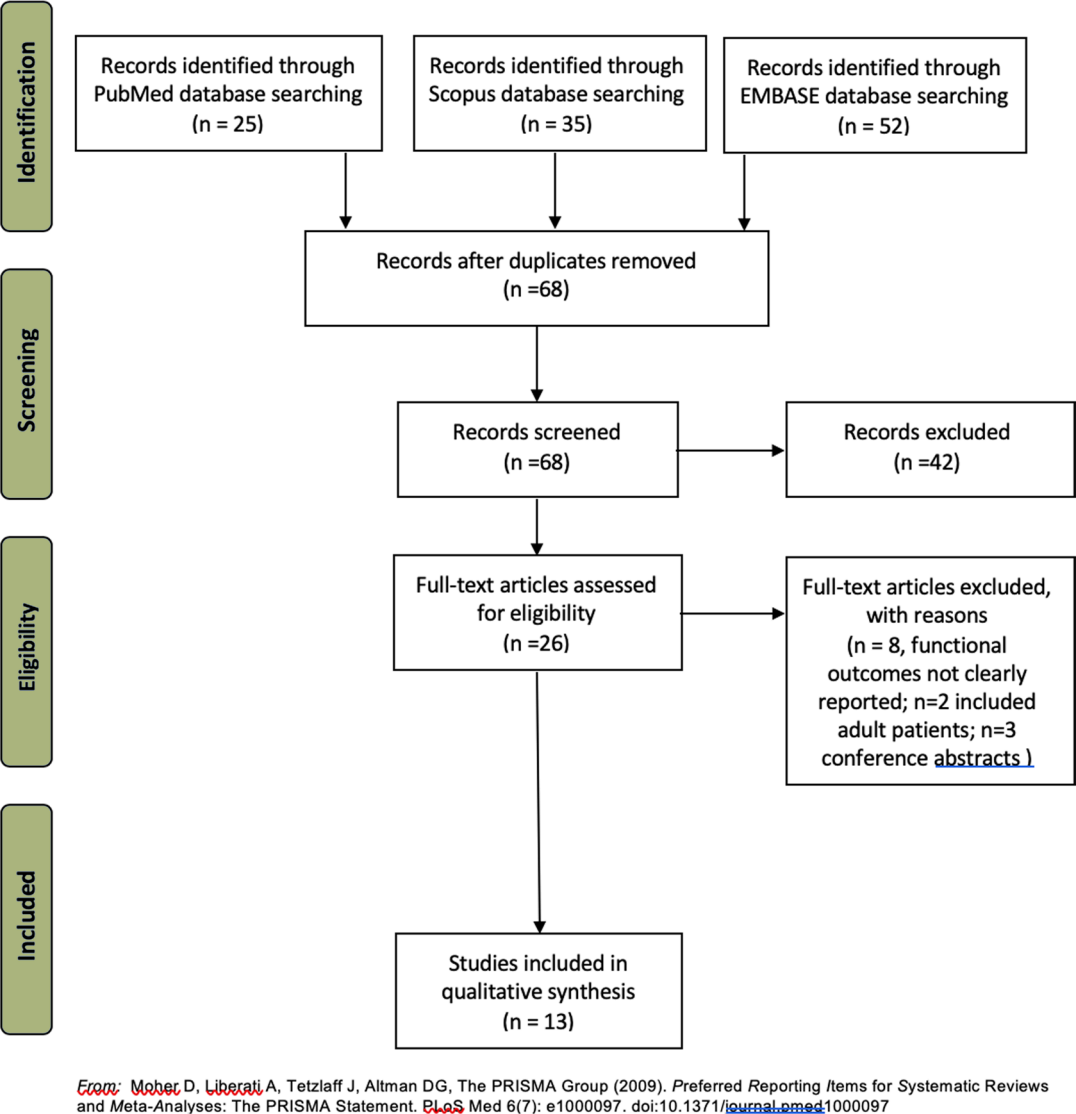
QUADAS-2 Score agreed upon two blinded reviewers. This tool assesses the risk of bias as low, high or unclear in four different parameters: patient selection, index test, reference standard and flow and timing.

Risk of bias was assessed by JL and IC, by using the tool developed by the University of Bristol known as QUADAS-2 (https://www.bristol.ac.uk/population-health-sciences/projects/quadas/quadas-2/). Any discrepancies were solved by discussion and agreement on a final punctuation. The bias assessment of each included study can be found in Figure 3.

## Results

### Identification of the studies

**Figure 4.**
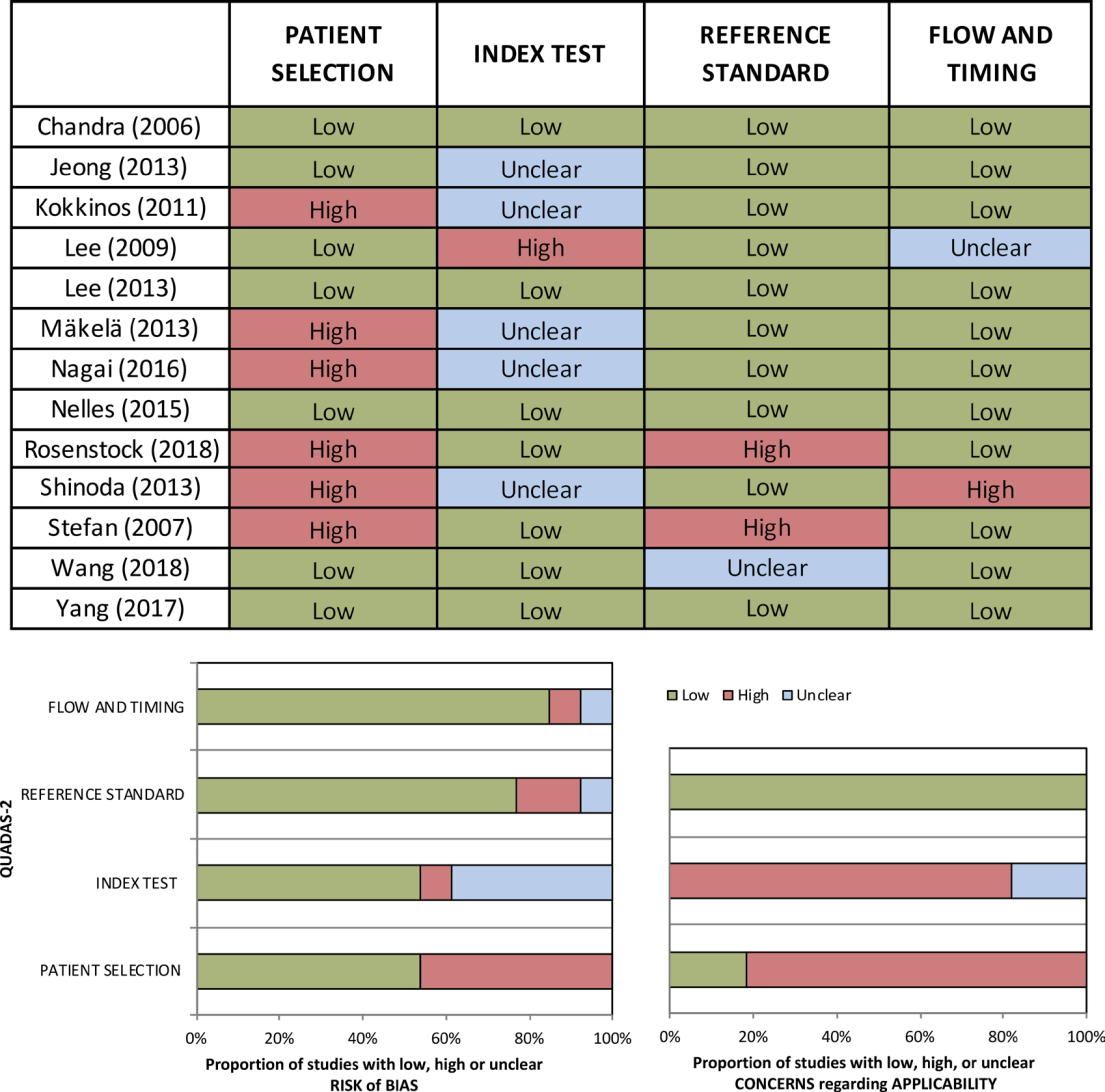
PRISMA flowchart of the selection process of the articles assessed for final analysis.^10^ PRISMA, Preferred Reporting Items for Systematic Reviews and Meta-Analyses.

The most common reasons for exclusion were, but not limited to, inclusion of an important proportion of adult patients within the study’s sample, no clear report of functional neurological outcome, no clear use of DTI or how DTI was used either for surgical planning and/or outcome prediction. A total of 13 studies (6 case reports, 5 retrospective studies, and 2 prospective studies) were finally included for qualitative synthesis since a meta-analysis was not possible due to the different outcomes reported. Figure 3 shows the results of bias assessment of each individual study. Figure 4 shows the flowchart of the selection process. Table 1 includes a summary of the main characteristics of the 13 included studies.

**Table 1.** Summary of the main characteristics of the 13 included studies

First author & year	Study design	Location	Sample size^*b*^	Age (Mean)	Pathology/Etiology	Seizure free	Imaging parameters
Stefan et al^40^ (2006)	Case Report	Germany	1 Female	12 years	Periventricular Nodular Heterotopia	Engel Class I	1.5 T Scanner Other parameters not reported
Kokkinos et al^41^ (2011)	Case Report	Greece	1 Male	10 years	Porencephalic Cyst	Engel Class I	1.5 T Scanner B-Value = 1000 s/mm^2^ Gradient Directions = 64
Nagai et al^42^ (2016)	Case Report	Japan	1 Male	2 years	Bilateral Unspecified Brain Malformation	Engel Class I	1.5 T Scanner B-Value = 700 s/mm^2^ Gradient Directions = 30
Rosentock et al^43^ (2018)	Case Report	Germany	1 Male	6 years	Left Thalamic DNT	Not Reported	Not Reported
Mäkelä et al^44^ (2013)	Case Series	Finland	1 Male	9 years	Type 2 FCD	Engel Class I	Not Reported
Shinoda et al^45^ (2013)	Case Series	Japan	2 Females	five and 10 years	DNT	Engel Class I	3.0 T Scanner Other parameters not reported
Chandra et al^46^ (2006)	Retrospective Cohort	U.S.A., India	15 (10 Females)	6.6 years	TSC	Engel Class 1 (9/11)	1.5 T Scanner B-Value = 1200 s/mm^2^ Gradient Directions = 6
Lee et al^47^ (2009)	Retrospective Cohort	Korea	27 (11 Females)	7.8 years	FCD (*n* = 9) Gliosis (*n* = 4) Nonspecific (*n* = 2) Microdysgenesis (*n* = 11) Encephalomalacia (*n* = 1)	Engel Class I (16/27)	Not Reported
Lee et al^48^ (2013)	Retrospective Cohort	Korea	72 (32 Females)	8.9 years	DNT (*n* = 4) Ganglioglioma (*n* = 3) Cortical Dysplasia (*n* = 32) Hippocampal Sclerosis (*n* = 9)^*a*^	Engel Class I (53/72)	3.0 T Scanner B-Value = 600 s/mm^2^ Gradient Directions = 32
Jeong et al^49^ (2014)	Retrospective Cohort	U.S.A.	31 (15 Females)	8.3 years	Not Reported. Only reported that 15 patients had a structural lesion.	Not Reported	3.0 T Scanner B-Value = 1000 s/mm^2^ Gradient Directions = 55
Wang et al^50^ (2017)	Retrospective Cohort	U.S.A., Canada	25 (14 Females)	8.3 years	Tumor (*n* = 2) Stroke (*n* = 12) Sturge-Weber (*n* = 1) Polymicrogyria (*n* = 2) Cortical Dysplasia (*n* = 3) Rasmussen Encephalitis (*n* = 2) Hemimegalencephaly (*n* = 3)	Not Reported	3.0 T Scanner B-Value = 1000 s/mm^2^ Gradient Directions = 10–30
Nelles et al^51^ (2015)	Prospective Cohort	Germany	29 (13 Females)	12 years	Stroke (*n* = 11) Pachygyria (*n* = 1) Polymicrogyria (*n* = 2) Hemiatrophy (*n* = 2) Hemimegalencephaly (*n* = 4) Rasmussen Encephalitis (*n* = 8) Postmeningitic Defect Zone (*n* = 1)	Engel Class I	3.0 T Scanner B-Value = 600 s/mm^2^ Gradient Directions = 16–32
Yang et al^20^ (2017)	Prospective Cohort	Australia	16 (8 Females)	9.8 years	FCD (*n* = 4) DNT (*n* = 5) Gliosis (*n* = 2) TSC (*n* = 3) Nonspecific (*n* = 1) Chronic Encephalitis (*n* = 1)	Engel Class I (12/16)	3.0 T Scanner, HARDI Sequence B-Value = 3000 s/mm^2^ Gradient Directions = 60

DNT, Dysembryplastic neuroepithelial tumor; FCD, Focal cortical dysplasia; TSC, Tuberous sclerosis complex.

a This study only reported the pathologic diagnosis in 52 patients that underwent complete epileptogenic resection. The remaining patients underwent corpus callosotomy (*n* = 18) and hemispherectomy (*n* = 2).

b Sample size included in the table includes only the data of pediatric patients (0–18 years of age) and not the total sample size of each study.

### Motor outcome

**Figure 5.**
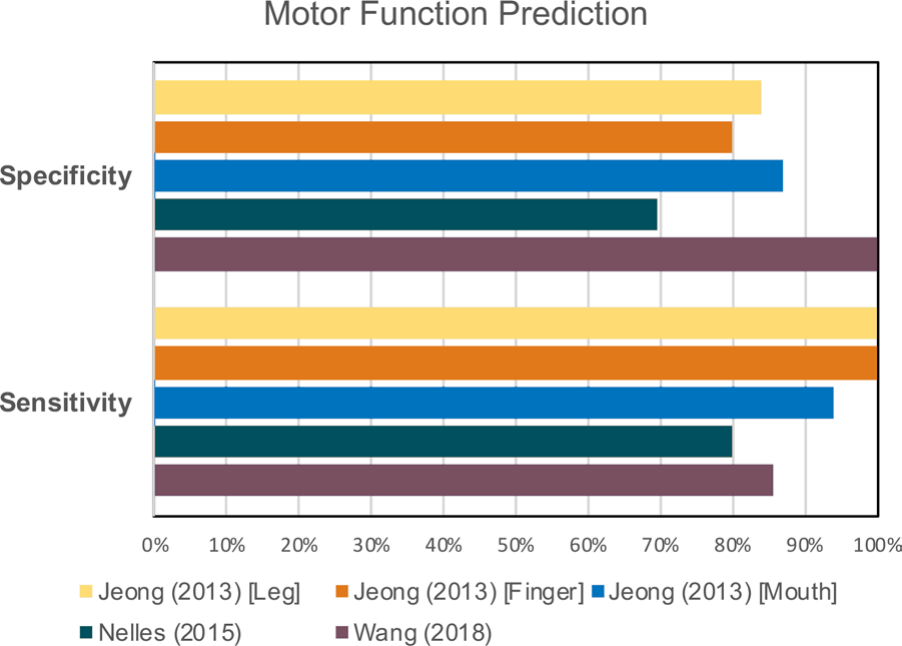
Pooled data regarding sensitivity and specificity of quantitative DTI values for prediction of post-operative motor outcome.^10^ DTI, diffusion tensor imaging.

Of the analyzed studies, seven reported the use of DTI and its relationship to the patient’s motor outcome.^20,41,45,48–51^ The studies by Kokkinos and Shinoda were case reports in which DTI was used to assess the relationship of the corticospinal tracts (CST) with the region of interest resulting in a tailored surgical plan to avoid this tract resulting in preservation of motor function in both patients..^41,45^ Yang et al reported on a prospective study analyzing 16 patients and using HARDI diffusion-weighted data which enabled a tailored surgical plan resulting in 100% preservation of post-operative motor function.^20^ Finally, the last three studies by Nelles, Jeong and Wang focused on the use of DTI for predicting motor outcome after epilepsy surgery and showed that quantitative values calculated with DTI, such as FA and the robustness of the CST, can accurately predict post-operative motor function with a specificity that ranges between 69.6 and 100% and a sensitivity of 80–85.7%^49–51^ (Figure 5).

### Language outcome

Three studies reported the use of DTI and its relationship with the patient’s language outcome.^41,43,48^ Two studies are case reports and the study by Lee and collaborators includes the report of different functional outcomes from which only five patients are reported as language outcome. A total of seven patients between the three studies and all the patients (100%) had complete preservation of language function after epilepsy surgery.

### Visual field outcome

Five studies reported the use of DTI and its relationship with the patient’s visual outcome.^20,40,41,45,48^ The total number of pooled patients is six, all the studies are case reports besides the one by Lee and collaborators that only reported the use of DTI to detect and preserve the OR in two patients. Only one patient had a superior quadrantanopia due to lesion of the Meyer’s Loop.^20^

### Lesion localization

Only one article reported the use of DTI in localizing epileptogenic tubers in 15 patients with the diagnosis of Tuberous Sclerosis Complex (TSC).^46^ Their hypothesis was that ADC would be increased in epileptogenic *vs* non-epileptogenic tubers. This retrospective study that included 15 pediatric patients assessed the usefulness of quantitative data obtained using both DWI and DTI and showed that highest ADC values had a sensitivity of 39.1%, specificity of 92.6% and accuracy of 86.5% for identifying epileptogenic tubers from those that didn’t caused seizures. ADC increased in epileptogenic *vs* non-epileptogenic tubers (*p* = 0.0031). Mean FA values showed no difference between epileptogenic or non-epileptogenic tubers (*p* = 0.77)..^46^

### Post-operative neurological status

10 of the 13 included studies reported post-operative seizure control reporting Engel Class I in 97/161 (60.2%).^20,40–42,44–48,51^ All of the analyzed studies report the post-operative neurological status of the patient in the following domains: motor outcome, language outcome and visual outcome. In the present study, neurological outcome has been classified as favorable when an improvement or maintenance of the pre-operative neurological function was reported; whereas unfavorable included reports of worsening neurological function. As shown on Table 2, be it either due to its qualitative utility in showing the anatomical relationships or its quantitative reporting for outcome prediction, DTI is related with a high degree of favorable neurological outcome.

**Table 2.** Table showing the pooled data of the analyzed studies that reported post-surgical neurological outcome

Neurological outcome			
Function	Favorable	Unfavorable	Total
Motor	75	33	108
Language	7	0	7
Visual	5	1	6
	87	34	121

## Discussion

This review showed that DTI can be useful in predicting and improving the motor outcome in pediatric patients after surgery. Our findings suggest that there are studies that confirm that quantitative DTI data can be used to predict the motor outcome after surgery, as well as studies that suggest that the use of DTI for pediatric epilepsy surgery is a useful tool to determine the course and localization of WMT and their relationships with the region of interest to be respected. Various of our included studies show that the use of DTI helps in creating a tailored plan that results in better functional outcome. However, caution is advised since the pool of patients included are heterogenous as well as the tractography techniques being used. A previous systematic review, published in 2016, reported the utility of DTI in pediatric epilepsy patients.^30^ However, this review was more focused on reporting the available studies on DTI, including even literature reviews and one systematic review, and assessing them through “rapid evidence assessment”, focusing more on reporting study design, quality, country of origin and general findings. As discussed before, their study included mixed pools of patients, adults and pediatric alike, and didn’t report the number of patients or their functional neurological outcome.^30^ Our review, on the other hand, only included the studies reporting, in its majority, pediatric patients as well as different functional outcomes including both neurological outcome and seizure freedom.

The major premise to epilepsy surgery is that any resection is undertaken with minimal or no adverse functional outcome. This is specifically relevant to motor function. While studies included here did show that quantitative DTI data can be used to predict the motor outcome after surgery,^49–51^ despite having a good number of patients (*n* = 85), as well as clear inclusion and exclusion criteria, they analyzed different parameters. For example, Jeong et al^49^ used a specific type of DTI to evaluate how fibre location related to those defined utilizing direct electrical stimulation of the brain and how resection in areas of higher density related to worse post-operative outcome. To the contrary, Nelles et al^51^ used the measurement of FA as a marker of post-operative motor outcome and reported a sensitivity of 80% and a specificity of 69.9% in predicting worse motor outcome. The role of DTI in the pre-operative planning of surgery is clearly stablished,^52–54^ however due to the lack of evidence and the different parameters assessed on the available evidence, more studies are desirable in order to determine the best surrogate marker from quantitative DTI data to predict post-operative motor outcome in pediatric epilepsy patients. Also, longer duration of follow-up period is required in order to assess whether motor strength remains after the initial post-surgical period, as well as whether patients with poor immediate motor outcome undergo some degree of recovery.

Preservation of existing language function and the potential for further development is obviously also important in children, despite the possibility of plasticity and reorganization. As already stated above, DTI in studies to date have been able to give the neurosurgeon the ability to create a tailored surgical plan for each patient which resulted in no language deficits after surgery.^41,43,48^ However, care must be taken when analyzing these data because a total number of seven patients is not enough to determine a clear prediction. Therefore, further studies with larger, consecutive and prospective patient cohorts are desirable in order to not only asses if DTI with reconstruction of the arcuate fasciculus is useful for guidance during epilepsy surgery, but also, as shown in the motor outcome section of these papers, assess whether there is any quantitative value of DWI or DTI such as FA, DWI map or ADC map that can be a useful predictor of post-operative language preservation.

DTI and 3D multimodality imaging (3DMMI) have been successfully used in adult patients to assess the location of the optic radiation and guide the neurosurgeon during epilepsy surgery.^54–56^ Nonetheless, as shown in this review, there is a lack of reporting of these techniques in children. The only studies found to report the use of DTI in identifying the OR are mostly case reports that amount to the small total of six patients. As already explained when discussing language outcome, this number is not enough to give any type of recommendation. Regardless, it is clear that the localization of the optic radiation via means of DTI is a useful procedure that could be implemented in all patients before epilepsy surgery that involves particularly the temporal lobe.^55^ More studies with a larger number of patients are needed to implement the use of DTI and show its usefulness in pediatric epilepsy surgery as well as determining the best parameters to be used for the tractography.

Finally, the study done by Chandra and collaborators used quantitative data of DWI and DTI to possibly assess and differentiate epileptogenic from non-epileptogenic tubers in 15 patients diagnosed with TSC. Despite the positive correlation of ADC values with identification, care must be taken since not all tubers were assessed in the same way and a larger cohort of patients would be desirable before ADC values could be safely used to distinguish between these lesions, considering such should be performed as part of a comprehensive presurgical evaluation.^46^

One such way, as has been already implemented in adults, could be the used of computational MRI analysis and the construction of 3DMMI maps that can be uploaded to a neuronavigational software that would enable the neurosurgeon to both plan the surgery and know exactly the area of resection while performing the surgery.^57–61^ Besides using it for planning the electrode’s trajectory, as most of the studies in adults patients have done, 3DMMI can also aid in lesion resection and can become particularly useful when the epileptogenic areas are not clearly demarcated by a lesion.^59^ In these circumstances, the neurosurgeon needs to operate in an area without clear anatomical boundaries, so the use of 3DMMI integrated with neurophysiological data can aid not only in the surgical planning, but also intraoperatively with the use of iMRI to confirm the area of resection and create 3D models beforehand that can be exported and used in the neuronavigational software as guidance during surgery.^59^ Certainly, the use of this type of software could aid in reducing the neurosurgical footprint in the brain by further integrating the data obtained by DTI which reconstructs the WMT that can be closely related to the region of resection.^14^ Multiple studies by Winston et al and Duncan et al have shown the usefulness of DTI in outcome prediction as well as surgical planning and intraoperative guidance during epilepsy surgery in adults,^22,23,62,63^ the acquisition of further data on utilization of such techniques in children is warranted. Certainly, DTI cannot be used as the sole imaging modality for pre-operative planning, but rather as another tool in an arsenal composed of different non-invasive imaging techniques that, through 3DMMI, can improve the functional neurological outcome in pediatric epilepsy patients.

In summary, our review has shown that the DTI can be a useful tool to determine the course and localization of WMT and help in creating a tailored plan for better functional outcome as reported by the included studies. However, due to the heterogeneity of the included patients as well as the tractography parameters, a categorical recommendation cannot be given. DTI seems to have an advantage in predicting post-surgical motor outcome, however, prospective studies with longer follow-up periods are desired in order to assess whether motor strength remains after the initial post-surgical period or if patients with poor immediate outcomes undergo some degree of recovery. Moreover, such studies might be also useful to determine surrogate markers for quantitative DTI in order to predict other functional outcomes such as language and vision preservation. Nonetheless, this review reveals that there is an unexplained lack of focus regarding tractography techniques in pediatric epilepsy patients and that the available studies don’t include the use of advanced acquisition techniques such as DSI, constrained spherical deconvolution, among others. Therefore, further studies including better techniques to ensure a low rate of false-positive fiber reconstruction are needed in order to obtain the full picture for its utilization among pediatric patients, as this is a desirable field of study because these types of techniques offer a non-invasive approach which is attractive in this type of patients.
